# Supplementary material for: Stacking Model‐Based Classifiers for Dealing With Multiple Sets of Noisy Labels
Source: Biom J. 2025 Mar 12;67(2):e70042. doi: 10.1002/bimj.70042 (PMC11898607; doi:10.1002/bimj.70042)
Supplement: Supplementary file 1 — Supporting Information [file BIMJ-67-e70042-s001.zip › density-based-ensemble-model-main/competitors_real_data/netlab-master/netlab-master/help/gpunpak.htm]

Netlab Reference Manual gpunpak


# gpunpak

## Purpose

Separates hyperparameter vector into components.

## Synopsis

```
net = gpunpak(net, hp)
```

## Description

`net = gpunpak(net, hp)` takes an Gaussian Process data structure `net` and
a hyperparameter vector `hp`, and returns a Gaussian Process data structure
identical to
the input model, except that the covariance bias
`bias`, output noise `noise`, the input weight vector
`inweights` and the vector of covariance function specific parameters
`fpar` have all
been set to the corresponding elements of `hp`.

## See Also

`gp`, `gppak`, `gpfwd`, `gperr`, `gpgrad`

---

**Pages:**
Index


---

Copyright (c) Ian T Nabney (1996-9)
